# Evolution of Epigenetic Regulation in Plant Reproduction

**DOI:** 10.3390/epigenomes10030048

**Published:** 2026-07-09

**Authors:** Vladimir Brukhin

**Affiliations:** 1 School of Natural and Environmental Sciences, Newcastle University, Newcastle upon Tyne NE1 7RU, UK; vbrukhin@gmail.com; 2 Laboratory of Embryology and Reproductive Biology, Komarov Botanical Institute, Russian Academy of Sciences, 2 Prof. Popov Str., St. Petersburg 197376, Russia

**Keywords:** evolution of plant reproduction, epigenetic regulation, DNA methylation, small RNAs, RNA-directed DNA methylation (RdDM), Polycomb Repressive Complex 2 (PRC2), chromatin remodeling, genomic imprinting, seed development, apomixis

## Abstract

Epigenetic regulation has played a fundamental role in the evolution of plant reproduction. Across more than a billion years, ancestral genome-defense mechanisms in early eukaryotes were progressively expanded, diversified, and repurposed throughout the green lineage. Streptophyte algae assembled the first plant-specific methylation and small RNA systems, providing pre-adaptations for terrestrial reproduction. In bryophytes and early vascular plants, these systems became integrated into gametophyte development, sporogenesis, and meiotic genome protection. Seed plants experienced substantial diversification and expansion of chromatin regulators and small RNA machinery, enabling increasingly sophisticated control of cone, ovule, and embryo development. Angiosperms underwent the most dramatic rewiring of epigenetic pathways, including gene-family diversification, subfunctionalization, and the emergence of genomic imprinting, endosperm-specific demethylation, and lineage-specific reproductive small RNAs such as phasiRNAs. Convergent solutions, including imprinting, meiotic transposable element (TE) silencing, and TE-derived regulatory elements, arose independently across lineages. Rather than reflecting the emergence of entirely new molecular machinery, these innovations illustrate repeated functional co-option and regulatory rewiring of deeply conserved epigenetic modules. Ecological and life-history pressures further shaped epigenetic diversification, linking environmental stress, mating systems, and domestication to reproductive epigenetic plasticity. Recent evidence further demonstrates that epigenetic plasticity underlies the recurrent evolution of alternative reproductive strategies such as apomixis and contributes to reproductive responses to environmental stress. Advances in comparative epigenomics, single-cell technologies, and epigenome editing are now providing unprecedented opportunities to reconstruct the evolutionary history of reproductive epigenetic pathways and to harness them for crop improvement. Together, these findings reveal epigenetic regulation as a dynamic, modular, and deeply evolvable framework that has repeatedly enabled reproductive innovation throughout plant evolution.

## 1. Introduction

Epigenetic regulation has become central to understanding how plant reproductive systems evolved, diversified, and adapted across more than a billion years of evolutionary history. DNA methylation, histone modifications, small RNA pathways, and chromatin-remodeling complexes form a multilayered regulatory architecture that controls genome stability, gene expression, and cell identity [1,2,3]. Although these mechanisms originated long before plants existed, their progressive integration into reproductive processes such as meiosis, gametophyte differentiation, fertilization, embryogenesis, and seed development was neither linear nor uniform. Instead, epigenetic systems were repeatedly reshaped by genome architecture, life-cycle innovations, and ecological pressures, producing lineage-specific solutions to the challenges of sexual reproduction [4].

The central problem addressed in this review is how ancient chromatin-based genome-defense systems were transformed into the highly specialized reproductive regulatory networks characteristic of modern land plants and especially angiosperms. Early eukaryotes evolved DNA methylation systems and RNA interference pathways primarily to suppress transposable elements (TEs) and maintain genome integrity [5]. The canonical RNA-directed DNA methylation (RdDM) pathway emerged later through the integration of these ancestral silencing mechanisms into plant-specific regulatory networks. Subunit homologs of Polycomb Repressive Complex 2 (PRC2), a suppressor of gene expression through triple methylation of histone H3K27, also originated in the ancient eukaryotes before their diversification [6]. As plants transitioned from aquatic streptophyte algae to terrestrial embryophytes, these ancestral systems were elaborated into plant-specific pathways such as CHROMOMETHYLASE-mediated methylation and canonical RdDM [7]. These innovations likely facilitated increasing developmental complexity, genome stability during meiosis, and the regulation of alternation of generations.

The rise of vascular plants (tracheophytes) and seed plants (spermatophytes) introduced new developmental contexts in which epigenetic regulation became essential. Massive increases in genome size, repetitive content, and developmental complexity, driven by transposable element (TE) proliferation and frequent whole-genome duplications (WGDs) created selective pressures for expanded silencing pathways and chromatin-remodeling capacity, specifically involving DNA methylation, histone modifications, and RNA-directed DNA methylation, to maintain genome stability and regulate gene expression [8,9]. In gymnosperms, which diverged in the late Devonian, strong DNA methylation and heterochromatinization evolved to stabilize massive TE-rich genomes, while early diversification of Argonaute (AGO), Dicer-like (DCL), and RNA-dependent RNA polymerase (RDR) families facilitated further evolution of small RNA pathways that later acquired specialized roles in reproductive development [10,11]. Angiosperms represent the most dramatic reorganization of epigenetic control in plant evolution. The emergence of highly reduced gametophytes and the mechanism of double fertilization (when one sperm cell fuses with the egg cell to form a diploid embryo (2n), while a second sperm cell fuses with the two polar nuclei (n) or the diploid central cell nuclei (2n) to form a triploid endosperm (3n)) are key innovations in angiosperms that generated distinct epigenetic asymmetries between maternal and paternal genomes [12,13,14,15,16]. Selective DNA demethylation in the central cell, paternal genome hypermethylation, and PRC2-mediated repression established the canonical form of genomic imprinting associated with endosperm development, a feature currently known only from angiosperms. In parallel, lineage-specific expansions of small RNA pathways, including reproductive phasiRNAs, introduced dynamic regulatory layers that fine-tune meiotic progression, gamete specification, and early embryogenesis [17,18].

The evolution of plants from aquatic streptophyte algae to modern angiosperms spans more than 500 million years and encompasses a series of major evolutionary transitions, including terrestrialization, the origin of mulicellular gametophytes, vascular tissues, the emergence of seeds, and the diversification of flowering plants [19,20]. Emerging technologies, including single-cell and spatial epigenomics, long-read methylome sequencing, and chromatin accessibility profiling, are now providing unprecedented resolution for understanding how epigenetic regulation operates during plant reproduction and provide opportunities to reconstruct the evolutionary history of epigenetic regulation across the green lineage. This review synthesizes current evidence on the origins, diversification, and functional integration of epigenetic mechanisms in plant reproduction. Particular emphasis is placed on the evolution of DNA methylation, chromatin regulation, and small RNA pathways; their roles in gametophyte development, fertilization, embryogenesis, and seed formation; and their contribution to major evolutionary innovations in plant reproductive biology. Although the majority of molecular insights into reproductive epigenetics derive from angiosperm model systems, a smaller but growing body of evidence from streptophyte algae, bryophytes, ferns, and gymnosperms provides important evolutionary context and is discussed throughout this review. However, the applicability of most mechanistic insights derived from angiosperm model systems to other plant lineages remains to be fully validated.

## 2. Origins and Evolution of Core Epigenetic Machinery

### 2.1. Proterozoic Origins: Ancestral Genome-Defense Systems

The core epigenetic systems that underpin plant reproduction today have deep evolutionary roots extending back to early eukaryotes. Comparative genomic analyses indicate that the Last Eukaryotic Common Ancestor (LECA), which lived approximately 1.5–1.8 billion years ago during the Proterozoic, possessed a sophisticated chromatin regulatory toolkit, including cytosine DNA methyltransferases (DNMTs), histone-modifying enzymes, ATP-dependent chromatin remodelers, and RNA interference (RNAi) machinery [21,22,23,24]. These ancient systems were likely associated primarily with genome surveillance and defense, particularly the suppression of transposable elements (TEs) and the maintenance of genome stability. Many components that later became integrated into plant-specific epigenetic pathways are also evolutionarily ancient. Homologs of AGO, DCL, RDR, and de novo DNA methyltransferase, DOMAINS REARRANGED METHYLTRANSFERASE (DRM) proteins, which form the core of RNA-directed DNA methylation (RdDM) in land plants, originated long before the emergence of embryophytes. Although the canonical RdDM pathway evolved later, its molecular foundations were already present in ancestral eukaryotic silencing systems [25,26]. Because meiosis creates periods of increased chromatin accessibility and potential transposon activation, early eukaryotes likely relied on epigenetic mechanisms to safeguard genome integrity during reproduction. Consequently, the association between epigenetic regulation and reproductive processes predates the origin of plants and provided the evolutionary foundation upon which lineage-specific reproductive epigenetic systems were subsequently built [25,26].

### 2.2. Streptophyte Algae: Assembly of Plant-Specific Epigenetic Modules

The divergence between Chlorophyta and Streptophyta represents a major evolutionary transition in the organization and regulation of green plant genomes. This split, which occurred more than one billion years ago, marked the beginning of a trajectory that ultimately led to the complex epigenetic systems characteristic of land plants. Although chlorophyte algae possess DNA methylation and RNA-silencing pathways, streptophyte algae, the closest algal relatives of land plants, exhibit a pronounced expansion and diversification of epigenetic regulatory mechanisms [27,28,29,30].

**Figure 1:**
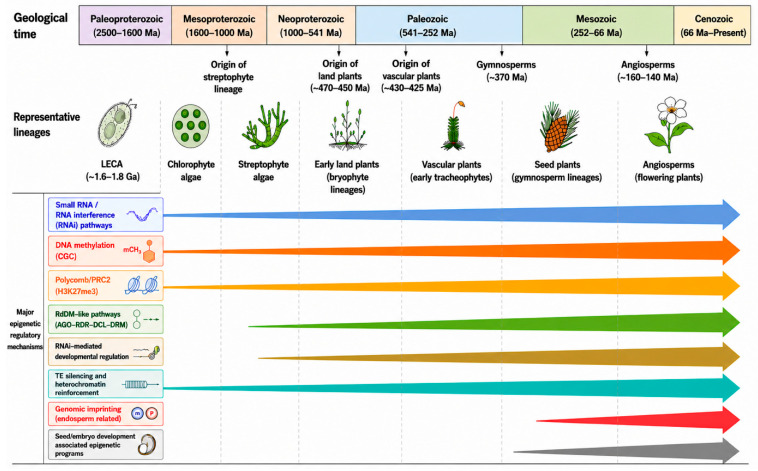
Evolutionary timeline of major epigenetic regulatory mechanisms across the green lineage. Schematic representation of the evolutionary history of major epigenetic pathways from the Last Eukaryotic Common Ancestor (LECA) to modern angiosperms. The horizontal axis spans 1.8 Ga (Ga (Giga-annum)—billion years ago) to the present, with key evolutionary transitions indicated: origin of land plants (~470–450 Ma), vascular plants (~430–425 Ma), gymnosperms (~370 Ma), and angiosperms (~160–140 Ma); Ma—millions of years ago. Dates indicate approximate ages of major evolutionary events based on current fossil and molecular evidence. Silhouettes represent major evolutionary grades: LECA, green algae, streptophyte algae (filamentous), early land plants, bryophytes, ferns, gymnosperms, and angiosperms. Colored arrows indicate the evolutionary assembly and increasing functional integration of major epigenetic regulatory systems. Arrow width represents the relative expansion, diversification, and functional importance of each pathway through evolutionary time rather than quantitative measurements. Blue arrow denotes small RNA/RNA interference (RNAi) pathways; orange arrow indicates DNA methylation systems; yellow arrow represents PRC2-mediated H3K27me3 regulation; green arrow indicates RNA-directed DNA methylation (RdDM)-like pathways; olive arrow denote RNAi-mediated developmental regulation; teal arrow represents reinforcement of transposable element (TE) silencing; red arrow indicates genomic imprinting; and dark gray arrow represents epigenetic regulation associated with seed development. The figure emphasizes that many components of the epigenetic machinery originated early in eukaryotic evolution, whereas their progressive recruitment into reproductive developmental processes occurred throughout plant evolution. Small RNA pathways, DNA methylation, and Polycomb-mediated chromatin regulation have deep evolutionary origins, with substantial expansion and functional specialization occurring in streptophytes and land plants. TE silencing and RNA-mediated developmental regulation became increasingly important during the evolution of vascular plants and seed plants. Canonical genomic imprinting is shown as an angiosperm innovation associated with the evolution of double fertilization and endosperm development. The diagram illustrates evolutionary trends and the relative timing of pathway integration rather than precise dates of origin or quantitative pathway activity. The earliest major radiation of seed plants inferred from current phylogenomic evidence and should not be interpreted as the crown-group age of extant seed plants.

Comparative genomic, epigenomic, and gene-family analyses indicate that several molecular components later central to plant reproductive epigenetics were already present in streptophyte algal ancestors. This includes ancestral homologs of SET domain histone methyltransferases, Polycomb components—functional PRC2, including homologs of E(z) and Su(z)12, AGO proteins, *DCL* genes and elements of DNA methylation machinery. Rather than emerging as fully integrated pathways, these regulatory modules likely evolved initially in distinct contexts, including chromosome organization, transposon repression, developmental regulation, and environmental stress responses, before becoming incorporated into reproductive developmental programs [28,31] (Figure 1).

Particularly important was the evolution of chromatin-based mechanisms capable of maintaining alternative developmental states. In early streptophytes, transitions between haploid and diploid phases were regulated by conserved developmental regulators such as TALE homeodomain proteins of the KNOX and BELL families. Increasing evidence suggests that these developmental circuits became progressively integrated with chromatin-level regulatory systems during streptophyte evolution [32]. Consistent with this view, PRC2-mediated repression plays a central role in life-cycle regulation in bryophytes, where it suppresses sporophytic developmental programs during the gametophyte phase [33]. In this sense, Polycomb-mediated silencing was first associated with life-cycle regulation before being recruited into increasingly specialized reproductive functions.

Genomic studies of *Klebsormidium*, *Zygnema*, and *Chara* reveal the presence of CHROMOMETHYLASE-like (CMT-like) methyltransferases associated with CHG methylation, a characteristic feature of land plant DNA methylation systems [34,35,36]. Similarly, the discovery of functional PRC2 components in Zygnematophyceae overturned the earlier assumption that Polycomb-mediated repression originated in embryophytes and instead demonstrated that this regulatory module predates the colonization of land.

Another important development was the expansion of small RNA pathways. Streptophyte algae produce 21–24 nt small interfering RNAs involved in transposon silencing, representing an early form of the RNA-mediated regulatory systems that later became central to epigenetic regulation in land plants [29]. In addition, several streptophytes exhibit gene-body methylation patterns resembling those of embryophytes, suggesting that mechanisms distinguishing constitutively expressed genes from transposon-rich heterochromatin were already emerging before terrestrialization [37,38]. Collectively, these findings indicate that streptophyte algae assembled many of the epigenetic modules that were later integrated into land-plant reproductive systems. Rather than originating abruptly with the emergence of embryophytes, key components of reproductive epigenetic regulation appear to have evolved gradually through the expansion, diversification, and functional integration of pre-existing chromatin, DNA methylation, and RNA-silencing pathways.

### 2.3. Early Land Plants: Integration into Gametophyte–Sporophyte Life Cycles

The transition to land during the Ordovician–Devonian (~470–360 million years ago (Ma)) brought new selective pressures that further shaped epigenetic evolution. Current phylogenomic analyses generally recover bryophytes and tracheophytes as sister clades, although the relationships among the three extant bryophyte lineages (mosses, liverworts, and hornworts) remain under active investigation. Bryophytes already possessed a largely complete and evolutionarily conserved DNA methylation toolkit, including MET1-like, CMT-like, and DRM-like methyltransferases, capable of maintaining CG, CHG, and limited CHH methylation [39,40]. These methylation systems played essential roles in sporogenesis, gametophyte development, and meiotic genome protection. In *Physcomitrium patens*, PRC2-mediated H3K27me3 regulates the transition from vegetative to reproductive development and is required for proper sporophyte patterning [33,41,42]. Bryophytes also deploy 21–24 nt siRNAs that silence TEs during meiosis in the sporophyte and during gamete formation in gametangia, demonstrating that small RNA-mediated regulation was already integrated into reproductive regulation at the dawn of land-plant evolution [43] (Figure 1, Table 1).

As land-plant genomes grew larger and more TE-rich, additional mechanisms evolved to ensure efficient access of methyltransferases to densely packed heterochromatin. An important innovation was the use of DDM1 (Decrease in DNA Methylation 1), a chromatin remodeler that removes linker histone H1 from compact heterochromatic regions. By transiently opening these otherwise inaccessible domains, DDM1 enables methyltransferases to maintain silencing across TE-rich genomic compartments—an essential requirement for genome stability, particularly in reproductive tissues [1]. DDM1-like chromatin remodelers are broadly conserved across land plants and may have acquired increasing importance as genomes expanded.

### 2.4. Diversification of CMT, RdDM, AGO/DCL/RDR Families

As vascular plants emerged, epigenetic systems diversified further. One of the most consequential developments was the expansion of the CMT family. CMT3 became specialized for CHG methylation, while CMT2 evolved to target CHH methylation in heterochromatin, particularly in TE-dense genomic regions [7]. This diversification created a multilayered methylation system capable of stabilizing increasingly large and TE-rich genomes. In parallel, the RdDM pathway emerged as a uniquely plant-specific innovation. Partial RdDM-like components predated land plants, whereas the canonical RdDM pathway, defined by the specialized RNA polymerases IV and V, RDR2, DCL3, and AGO4/6/9, forming a small RNA-guided DNA methylation system that became essential for TE silencing in reproductive tissues, originated in seed plants and became fully elaborated in angiosperms [44] (Table 1). The *AGO*, *DCL*, and *RDR* gene families underwent repeated expansions across land-plant evolution, with major radiations in seed plants and angiosperms. These expansions enabled functional specialization, including the emergence of small RNA pathways that regulate gametogenesis and early embryogenesis [45] (Figure 1).

Complementing these methylation pathways, plants also evolved active DNA demethylation systems that allow targeted removal of methylation marks. This process is mediated by a family of DNA glycosylases, ROS1, DME, DML2, and DML3, which excise methylated cytosines and replace them with unmethylated bases via base-excision repair. These enzymes provide a counterbalance to methyltransferase activity and enable dynamic remodeling of methylation landscapes during reproduction and subsequent development [46,47]. Their activity ensures that regulatory loci can be selectively demethylated in response to developmental cues, thereby supporting flexible control of gene expression in reproductive tissues.

### 2.5. PRC2 Evolution: From General Repression to Reproductive Specialization

PRC2 also underwent significant evolutionary refinement. Although its core components are conserved across eukaryotes, plants evolved lineage-specific PRC2 complexes with distinct developmental roles. In bryophytes, PRC2 plays a critical role in regulating the haploid–diploid transition and sporophyte development, acting as an epigenetic silencer that prevents the premature development of the diploid sporophyte phase from haploid gametophytes; in ferns and gymnosperms, it contributes to sporangium differentiation and embryo patterning [6,48]; and in angiosperms, PRC2 diversified into specialized complexes such as FIS-PRC2, which is essential for endosperm development and genomic imprinting [49,50]. This progressive specialization illustrates how an ancient chromatin-repression system was repeatedly adapted to meet the regulatory demands of increasingly complex reproductive structures.

### 2.6. Whole-Genome Duplications and TE Load as Evolutionary Drivers

Whole-genome duplications (WGDs) and transposable element expansion are key drivers of plant evolution, providing raw genetic material for the evolution of complex, tissue-specific regulatory networks. These events often enable subfunctionalization, where duplicate gene copies (such as those in the RNA-directed DNA methylation pathway) share the ancestral functions, allowing for increased regulatory complexity and the emergence of specialized functions in reproductive development [45,51,52]. At the same time, transposable element proliferation, particularly in gymnosperms and angiosperms, created a strong evolutionary demand for robust methylation and small RNA pathways. Conifer genomes, which can exceed 20–30 Gb and whose big size is largely driven by the accumulation of long terminal repeat retrotransposons (LTR-RTs), exhibit exceptionally high levels of CG and CHG methylation, expanded CMT lineages, and extensive heterochromatinization, reflecting the evolutionary interplay between genome size, transposable element activity, and epigenetic silencing capacity [53,54].

Together, whole-genome duplications and transposable element expansion repeatedly generated new regulatory substrates upon which epigenetic mechanisms could evolve, facilitating the increasing complexity of plant reproductive systems. Ancient genome-defense systems were progressively elaborated in streptophyte algae, co-opted and integrated into reproductive development in early land plants, diversified in vascular plants, and specialized in seed plants. This evolutionary trajectory established the regulatory foundation upon which the complex epigenetics regulating reproductive processes in angiosperms would later be built (Table 1).

## 3. Evolution of Epigenetic Regulation in Gametophytes and Functional Germlines

The evolution of plant reproduction has been tightly coupled with the diversification of epigenetic mechanisms that regulate developmental transitions, genome stability, and transmission of heritable information. This relationship is particularly evident in gametophytes and germlines, where epigenetic regulation integrates developmental patterning with protection of genomic integrity during reproduction. Comparative and functional studies increasingly indicate that many of the pathways controlling reproductive epigenetic states originated early in green plant evolution and were progressively modified during the transition from ancestral streptophyte algae to land plants, seed plants, and angiosperms. Epigenetic mechanisms operating in reproductive lineages appear to have evolved through gradual recruitment and specialization of ancient chromatin-based regulatory systems (Figure 1) [28].

### 3.1. Bryophytes and the Emergence of Epigenetic Regulation of Alternating Generations

One of the earliest evolutionary contexts in which epigenetic regulation acquired significant importance was the control of life-cycle phase transitions. Bryophytes preserve key features of ancestral reproductive epigenetic organization. Because these non-vascular plants are haploid-dominant, they bypass the masking effects of diploidy and provide important insight into early functions of epigenetic regulation in reproduction [55,56]. Alternation of generations required robust mechanisms for maintaining the developmental identities of haploid and diploid phases, and comparative evidence suggests that chromatin-mediated repression played a key role in this process. Studies in bryophytes have shown that components of the PRC2, including homologs of *CURLY LEAF* (*CLF*), *SWINGER* (*SWN*), and *FERTILIZATION-INDEPENDENT ENDOSPERM* (*FIE*), contribute to repression of sporophytic developmental programs during gametophyte growth, indicating an ancient role for H3K27me3-mediated silencing in life-cycle regulation [33,57,58]. The pre-embryophytic origin of PRC2 has been convincingly demonstrated in mutants with reduced PRC2 activity, and the role of PRC2 has been analyzed in extant species in the Archaeplastida lineage and in the diatom *P. tricornutum* [58]. In *Physcomitrium* and *Marchantia*, disruption of PRC2 components including *CLF*, *FIE*, and related factors causes ectopic activation of sporophytic developmental programs in gametophytic tissues, indicating that Polycomb repression became central to maintaining generation identity [33,41]. This may represent one of the earliest examples where chromatin regulation was transformed from general developmental control into reproductive phase specification. Thus, an important innovation at this evolutionary stage was the integration of epigenetic regulation with alternation of generations itself.

Bryophytes also reveal early coupling between heterochromatin regulation and reproductive function [59]. Components related to CMT, methyltransferase SUVH4/KYP (KRYPTONITE), and RNA-mediated silencing contribute to transposon repression in gametophytic tissues, suggesting that germline-associated genome-defense systems were already being elaborated in early land plants [40]. Importantly, this period likely saw the first major transformation of epigenetic systems from largely genome-stabilizing functions by silencing repetitive DNA toward developmental specialization and chromatin pathways became integrated with regulation of reproductive identity, a fundamental evolutionary step.

### 3.2. Vascular Plants and the Diversification of Reproductive Epigenetic Modules

The evolution of vascular plants introduced major changes in reproductive architecture, including heterospory, reduction of free-living photosynthesizing gametophytes, and increasing separation of reproductive developmental programs. In vascular plants, unlike bryophytes, the predominant phase is the sporophyte. These transitions reshaped selective pressures acting on reproductive epigenetic systems. Reduction of gametophytes intensified demands for developmental canalization and functional germline protection, favoring greater specialization of chromatin and methylation pathways. This period likely saw substantial diversification of DNA methylation systems, including functional partitioning among *MET1*, *CMT3*, and *DRM*-related pathways. Evidence also suggests that the RNA-directed DNA methylation (RdDM) pathway acquired increasing importance during seed plant evolution [60,61] (Table 1). While core components of the RNA-directed DNA methylation pathway—such as RNA-dependent RNA polymerase 2 (RDR2), ARGONAUTE 4 (AGO4), and DICER-LIKE 3 (DCL3)—originated earlier in plant evolution and exist in ancient plant lineages like bryophytes (mosses) and non-flowering vascular plants, comparative analyses indicate that the canonical Pol IV/V-dependent RdDM pathway became fully elaborated in seed plants, particularly in angiosperms [62]. This represents a second major evolutionary transformation: epigenetic pathways originally centered on transposon silencing became expanded into developmental regulatory systems increasingly integrated with reproduction. Small RNA evolution also accelerated during this period. Expansion of *AGO* and *DCL* families provided substrates for functional divergence, with some paralogs retaining ancient silencing roles while others acquired specialized reproductive functions. Such duplication-driven partitioning was a recurrent mechanism in epigenetic evolution [45]. Importantly, these patterns reveal an apparent contradiction between deep conservation of epigenetic machinery and strong lineage-specific divergence in reproductive function. This suggests that evolutionary change occurred primarily through functional rewiring of conserved pathways rather than through the emergence of novel mechanisms. In this context, epigenetic systems should be viewed as evolutionarily flexible modules whose regulatory deployment, rather than biochemical identity, underwent major diversification across plant lineages.

### 3.3. Seed Plants: Genome Conflict and Reproductive Epigenetic Innovation

The origin of seeds introduced new evolutionary challenges—including prolonged reproductive development, parental genome interactions, and increased transgenerational demands that profoundly reshaped epigenetic regulation. Evidence suggests that the capacity for reproductive methylation reprogramming evolved alongside the increasing complexity of plant reproduction. Extensive central-cell-specific DNA demethylation and endosperm-associated reprogramming are characteristic features of angiosperms, and their emergence appears to coincide with the evolution of double fertilization and the triploid endosperm, a developmental innovation that originated roughly 140–125 million years ago. More limited forms of reproductive methylation dynamics, however, have also been reported in other land-plant linages [4,63].

The expansion of transposable elements (TEs) in seed plant genomes created a form of “genomic shock” that imposed strong selective pressure for the evolution of increasingly sophisticated epigenetic silencing mechanisms. This pressure likely contributed to the diversification of the RNA-directed DNA methylation (RdDM) pathway into a highly specialized surveillance system. At this stage, epigenetic regulation expanded beyond genome defense to assume direct roles in reproductive development [64]. Likewise, Polycomb complexes, originally associated with general developmental repression, acquired increasingly specialized reproductive functions. In angiosperms, diversification of MEA, FIS2, and related PRC2 components enabled the evolution of novel regulatory mechanisms controlling endosperm and embryo development [65].

A key evolutionary innovation in seed plants may have been selective epigenetic reprogramming rather than genome-wide resetting. Unlike animals, plants generally preserve global DNA methylation patterns across generations while employing targeted, locus-specific methylation reprogramming in reproductive companion cells. This strategy provides developmental flexibility while maintaining long-term genome stability [66]. Active DNA demethylation is mediated by DEMETER (DME) and its related DNA glycosylases which regulate gene expression primarily in the central cell of the female gametophyte and the vegetative cell of the male gametophyte. Although these enzymes are evolutionarily ancient, their highly specialized role in companion-cell-specific DNA demethylation appears to have evolved in flowering plants [46,67].

### 3.4. Angiosperm Innovations: Germline Specialization and Epigenetic Asymmetry

In angiosperms, reproductive epigenetic regulation underwent further transformation through cell-type specialization and the multicellular organization of silencing mechanisms. One of the most significant innovations was the evolution of an epigenetic division of labor between germ cells and accessory companion cells. In pollen, partial relaxation of epigenetic silencing in the vegetative nucleus generates small RNAs that reinforce transposon repression in sperm through pathways involving Pol IV, RNA-dependent RNA polymerase 2 (RDR2), DCL3/4, and AGO4/6. This system, known as accessory companion cell regulation, represents not simply an extension of ancestral silencing mechanisms but rather a derived reorganization of epigenetic function across interacting cell types. Its evolutionary significance is considerable, as it transformed genome protection from a largely cell-autonomous process into an intercellular reproductive surveillance system, representing a major conceptual shift in the organization of epigenetic regulation [68,69].

Angiosperms also evolved lineage-specific reproductive small RNA systems, particularly phasiRNAs, which are largely absent in bryophytes and early vascular plants, first appeared in seed plants, and diversified extensively in angiosperms, especially monocots. These small RNAs are generated from long non-coding RNA precursors in a highly coordinated manner, with distinct size classes accumulating at different stages of anther development to ensure proper pollen formation. Their emergence coincided with the evolution of increasingly complex anther architecture and more refined regulation of male fertility [70,71]. In crops such as rice and maize, phasiRNAs accumulate during pre-meiotic and meiotic stages of anther development and are essential for normal male reproductive development. Although phasiRNAs are detected in all anther cell types, they accumulate most abundantly in tapetum and are thought to move from tapetal cells into meiocytes [72]. Mutations in key components of the phasiRNA biogenesis, including *DCL4*, *DCL5*, or *RDR6*, disrupt phasiRNA production and frequently result in male sterility, underscoring their critical developmental functions [73,74]. In addition to phasiRNAs, miR172 functions as a key post-transcriptional regulator of floral development in *Arabidopsis thaliana*. It controls floral organ identity and fertility by repressing *APETALA2* (*AP2*) and related *AP2-like* transcription factors (e.g., *TOE1*, *TOE2*, *SMZ*, *SNZ*). During floral transition, increasing miR172 expression downregulates these targets, thereby promoting normal reproductive development [75,76].

Another major innovation was the establishment of pronounced epigenetic asymmetry between male and female reproductive lineages. Female gametophytes evolved specialized DNA demethylation dynamics, companion-cell functions, and imprinting-associated regulatory mechanisms, whereas male gametophytes developed distinct chromatin-remodeling processes and small RNA-mediated genome surveillance systems. This functional asymmetry laid the foundation for later innovations, including parent-of-origin-specific gene expression and endosperm-associated epigenetic regulation [77,78].

Rather than representing isolated evolutionary novelties, these innovations illustrate the progressive restructuring of ancient epigenetic modules into highly specialized regulatory systems governing angiosperm reproduction.

### 3.5. Evolutionary Transformation of Functional Germline Protection Systems

An overarching pattern in plant evolution is the progressive transformation of germline protection systems. In early green lineages, genome surveillance was primary centered on basic transposon repression. In bryophytes and other early land plants, these pathways became increasingly integrated with the regulation of phase identity and reproductive development. In vascular and seed plants, the expansion of silencing machinery, TE-driven genome evolution, and gene-family diversification transformed these pathways into increasingly specialized reproductive regulatory networks. In angiosperms, additional innovations further reorganized these systems through intercellular signaling, selective epigenetic reprogramming, and lineage-specific small RNA pathways. This evolutionary trajectory shows that germline protection did not simply become stronger over time; rather, it became integrated into developmental regulation and increasingly capable of generating evolutionary novelty. Much of this history was driven by gene family evolution. Repeated expansion of *AGO*, *DCL*, *RDR*, *CMT*, *SUVH*, and PRC2 families created new opportunities for functional specialization (Figure 1; Table 1). Whole-genome duplications and subsequent gene retention provided paralogs that were repeatedly subfunctionalized and neofunctionalized, thereby increasing the complexity of reproductive epigenetic networks, particularly in seed plants.

Thus, the evolution of epigenetic regulation in gametophytes and germlines was not peripheral to plant reproductive evolution; it was one of its mechanistic foundations. From streptophyte ancestors to flowering plants, reproductive diversification was accompanied by progressive transformation of epigenetic systems, linking genome defense, developmental regulation, and evolutionary innovation into a single evolutionary continuum. Taken together, these comparisons reveal a clear pattern: early-diverging lineages primarily utilize epigenetic systems for genome defense and life-cycle regulation, whereas seed plants and especially angiosperms progressively co-opted these mechanisms for increasingly complex developmental and reproductive functions. This transition from conserved biochemical machinery to diversified regulatory roles underpins the emergence of lineage-specific reproductive strategies.

## 4. Fertilization, Embryogenesis, and Seed-Associated Epigenetic Innovations

The evolution of double fertilization, embryogenesis, and seeds in angiosperms necessitated a sophisticated epigenetic landscape to manage the complex developmental, nutritional, and protective processes underlying seed formation. This major evolutionary transition required novel regulatory mechanisms to establish distinct embryo and endosperm identities, coordinate parental genome interactions, and control nutrient allocation and seed maturation. Although many chromatin-based pathways involved in these processes originated much earlier during the evolution of the green lineage, the emergence of seed plants, and especially angiosperms, imposed new selective pressures associated with zygotic genome activation, parental genome interactions, embryo patterning, transposable element control, nutrient allocation, and developmental dormancy [79]. Comparative studies indicate that extensive DNA methylation during reproduction is a derived feature of angiosperms and is largely absent from earlier diverging land-plant lineages. In the moss *Physcomitrium patens*, for example, DNA methylation patterns remain largely stable throughout reproductive development and lack the extensive remodeling characteristic of angiosperm [80]. Similarly, gymnosperms generally maintain relatively stable DNA methylation levels during reproduction, exhibiting only modest changes in reproductive tissues, in contrast to the extensive methylation reprogramming observed in angiosperms. These observations suggest that large-scale, dynamic methylation remodeling associated with seed development is a comparatively recent evolutionary innovation of angiosperms rather than a universal feature shared by all seed plants [62,81].

### 4.1. Origins of Epigenetic Control in Zygotic Transitions

The epigenetic challenges associated with fertilization arose long before the origin of seeds. In the earliest eukaryotes, syngamy itself created the need to coordinate genome merging, chromatin reorganization, and the resetting of developmental programs. In green algae, although embryogenesis in the land-plant sense had not yet evolved, transition from gamete fusion to the diploid phase required primitive chromatin-based regulatory mechanisms. Comparative molecular evidence suggests that the regulatory logic underlying early zygotic activation in plants shares deep evolutionary roots with pathways controlling haploid–diploid phase transitions (alternation of generations) in ancestral algae. This relationship suggests that regulatory systems originally responsible for switching between gametophytic and sporophytic developmental programs were later co-opted to control embryogenesis. *TALE* homeodomain transcription factors, including *KNOX* and *BELL*, which are conserved across chlorophytes and streptophytes, were among the earliest regulators of post-fertilization developmental reprogramming. Their subsequent integration with chromatin-based regulatory pathways likely represented a key step in the evolution of epigenetic control over embryogenesis [82].

The emergence of multicellular embryos (the young sporophyte) in bryophytes, often referred to as the embryophytic habit or “maternal embrace”, represents one of the in land-plant evolution. This transition from the unicellular zygote of ancestral streptophyte algae to a multicellular embryo required mechanisms that stabilized developmental identity and coordinated cell differentiation, including epigenetic regulation [83,84]. Comparative studies suggest that the PRC2 complex was already involved in restricting embryo-associated developmental programs early in land-plant evolution (Figure 1; Table 1). The presence of conserved homologs of *CURLY LEAF* (*CLF*), *SWINGER* (*SWN*) and *FERTILIZATION-INDEPENDENT ENDOSPERM* (*FIE*) indicate that H3K27me3-mediated regulation of embryonic development programs has deep evolutionary origins [57].

This represents an important evolutionary shift, whereby chromatin-based regulatory systems that originally involved in maintained generation identity were co-opted to control embryogenesis itself. Such functional repurposing may have been fundamental to the origin of the multicellular plant embryo as a key evolutionary innovation.

### 4.2. Evolution of Epigenetic Regulation During Embryogenesis

Embryogenesis imposed new regulatory demands by requiring the progressive stabilization of cell fate, suppression of transposable element activity, and coordination of maternal and zygotic programs. Many of the epigenetic mechanisms underlying these processes evolved through the gradual functional diversification of pre-existing genome-defense pathways. DNA methylation acquired increasingly specialized roles during the evolution of vascular plant. Whereas ancestral methylation systems primarily maintained genome stability, maintenance methylation mediated by *MET1*, chromomethylation by *CMT3*, and de novo methylation catalyzed by *DOMAINS REARRANGED METHYLTRANSFERASE 2* (*DRM2*) became progressively integrated into embryo patterning, developmental robustness, and TE silencing [5,85,86]. In angiosperms, embryonic DNA methylation is characterized by selective, locus-specific remodeling while global CHG and CG methylation patterns are largely maintained across generations. This contrasts sharply with the genome-wide epigenetic reprogramming characteristic of mammals and represents a distinctive evolutionary strategy in which developmental progression is coordinated with effective transposon repression without erasing inherited epigenetic information [4]. As discussed above, extensive, cell type-specific methylation reprogramming appears to be a derived feature of angiosperm reproduction rather than a universal characteristic of seed plants.

Chromatin remodeling factors also became central regulators of embryogenesis. *DDM1*, *PICKLE* (*PKL*), and ATP-dependent chromatin remodelers facilitate chromatin-state transitions during embryo development, while the regulated deposition of histone variants contributes to developmental competence [87,88]. Particularly important was the evolutionary diversification of Polycomb Repressive Complex 2. Although ancestral PRC2 functioned broadly in developmental repression, it acquired increasingly specialized roles in embryo patterning, endosperm formation, and developmental timing during seed-plant evolution. In *Arabidopsis*, *CLF*, *EMF2*, *FIE*, and *MSI1* regulate chromatin-based repression networks essential for embryogenesis and seed development, illustrating the extensive functional diversification of this ancient regulatory complex [89,90].

Collectively, these innovations reflect a broader evolutionary transformation in which epigenetic systems evolved from maintaining developmental states primarily for genome stability to actively orchestrating embryonic development and ensuring robust embryo formation.

### 4.3. Seed Evolution and the Rise of Novel Epigenetic Demands

The origin of seeds marked one of the most profound shifts in reproductive evolution and fundamentally reshaped the landscape of epigenetic regulation. Seeds introduced prolonged developmental arrest, complex maternal–offspring interactions, nutrient allocation conflicts, and dormancy, all of which required new mechanisms of developmental memory and regulatory stability. These innovations imposed strong selective pressures for epigenetic systems capable of integrating developmental and environmental signals over long timescales.

The evolution of PRC2 appears to have been closely associated with these innovations. The FIS-class PRC2 complex, comprising the above-mentioned *MEA*, *FIS2*, *FIE*, and *MSI1*, represents a major reproductive innovation in seed plants. Although derived from an ancient Polycomb machinery, this complex acquired specialized functions in seed development and, in angiosperms, became essential for the regulation of endosperm cell division and genomic imprinting [89]. Its emergence represents one of the clearest examples of functional co-option during reproductive evolution, whereby a deeply conserved chromatin repression system was repurposed to regulate seed-specific developmental processes.

Seed evolution also strengthened the connection between epigenetic regulation and genome conflict. The expansion of transposable elements in seed plant genomes increased the selective pressure for robust DNA methylation and RNA-mediated silencing pathways. The diversification of key components of RdDM pathway—including *NRPD1*, *RDR2*, *DCL3*, *AGO4*, and *DRM2*—may have been driven, at least in part, by these evolutionary pressures [62].

### 4.4. Double Fertilization, Imprinting, and Epigenetic Asymmetry in Angiosperms

Among the most remarkable epigenetic innovations in plant evolution is the emergence of genomic imprinting, which is closely associated with double fertilization and endosperm evolution in flowering plants. Genomic imprinting, characterized by parent-of-origin-specific gene expression, is a hallmark of angiosperm endosperm development. It arises from differential epigenetic marking of maternal and paternal alleles involving DNA demethylation, histone modifications, and small RNA pathways [91]. In *Arabidopsis DEMETER* (*DME*) specifically expressed in the central cell of the female gametophyte, where it initiates active DNA demethylation by excising of 5-methylcytosine (5mC), leading to a hypomethylated maternal genome before fertilization. DME-like glycosylases are highly specialized regulators of seed development and genomic imprinting [92]. Maternal expression of *MEA*, *FWA*, and *FIS2* is established through DME-mediated DNA demethylation and subsequently maintained by the FIS-PRC2 complex, which represses paternal alleles and prevents premature endosperm proliferation [50]. An example of paternally imprinted gene in the *Arabidopsis thaliana* endosperm is *PHERES1* (*PHE1*) [93]. Although imprinting is widespread among angiosperms, the identities of imprinted genes differ substantially between species such, including *Arabidopsis* and rice, suggesting repeated lineage-specific evolution rather than strict conservation [94].

The parental conflict theory proposes that genomic imprinting evolved from divergent maternal and paternal interests over resource allocation to the developing embryo [95]. Excessive paternal gene activity can promote overproliferation of the endosperm and seed failure, whereas excessive maternal repression may restrict embryo growth and reduce seed fitness. The apparent absence of canonical genomic imprinting in gymnosperms and bryophytes supports the view that imprinting, as currently understood, evolved in association with the origin of the biparental endosperm and is best documented in angiosperms. Although parent-of-origin effects have been reported in gymnosperms, convincing evidence for canonical genomic imprinting remains lacking. However, the discovery of paternal chromosome repression in *Marchantia polymorpha*, where the paternal genome is globally silenced during sporophyte development, suggests that imprinting-like mechanisms may have evolved independently multiple times during plant evolution [96]. This observation supports the hypothesis that genomic imprinting arose through the repeated co-option of ancient TE-silencing pathways for reproductive developmental functions rather than from a single evolutionary origin [97].

Although genomic imprinting is well characterized in angiosperm endosperm, its evolutionary origin remains debated. One possibility is that imprinting evolved as a direct consequence of double fertilization and origin of the endosperm. Alternatively, it may represent a specialized manifestation of more ancient parent-of-origin asymmetries that predated flowering plants. The considerable variation in the identity of imprinted genes among angiosperm lineages suggests that imprinting is evolutionarily labile, with both imprinted loci and their underlying regulatory networks being repeatedly gained, modified, and lost during plant evolution.

### 4.5. Small RNA-Mediated Regulation of Embryogenesis and Seed Evolution

The emergence of small RNA-mediated genome regulation was an important innovation in the evolution of land-plant reproduction, providing a mechanism to stabilize the genome during the vulnerable phases of embryogenesis and seed development. Across embryophytes, small RNAs act as the primary regulators of transposable elements (TEs), and their diversification in seed plants, particularly angiosperms, enabled increasingly sophisticated regulation of reproductive development, parental genome interactions, and the maintenance of seed viability.

In seed plants, the RNA-directed DNA methylation (RdDM) pathway constitutes the central small RNA system responsible for TE silencing during reproduction. RdDM relies on 24-nt siRNAs generated by RNA polymerase IV, RDR2, and DCL3, which guide AGO4/6/9 to nascent scaffold transcripts produced by RNA polymerase V. This pathway is strongly upregulated in reproductive tissues, reflecting the high demand for TE suppression during gamete formation, fertilization, and early embryogenesis [98,99].

The relationship between transposable elements and reproductive evolution extends beyond genome defense. Increasing evidence suggests that TE-associated epigenetic divergence can contribute to hybrid incompatibilities, abnormal seed development, and reproductive isolation. These observations raise the possibility that TE silencing pathways are not merely responses to genome instability but may themselves influence speciation by generating lineage-specific epigenetic landscapes. Whether TE-driven epigenetic divergence represents a major mechanism of reproductive isolation across plant linages remains an important unresolved question.

A defining feature of angiosperm reproduction is the asymmetric deployment of small RNAs in the male gametophyte. In pollen, the vegetative cell undergoes partial DNA demethylation and controlled TE activation, producing a burst of 21–24 nt siRNAs that move into the sperm cells to reinforce TE silencing in the male germline [68,100]. This intercellular small RNA transfer is unique to flowering plants and represents an evolutionary solution to the challenge of protecting the haploid genome while maintaining rapid gametophyte development. The resulting reinforcement of TE repression in sperm contributes directly to embryo genome stability after fertilization.

In the female gametophyte, the central cell exhibits targeted DNA demethylation prior to fertilization, generating maternal siRNAs that shape genomic imprinting and early endosperm development [101,102]. These maternal siRNAs preferentially silence paternal alleles of TE-adjacent genes, establishing parent-of-origin-specific expression patterns that regulate nutrient allocation and endosperm proliferation. The maternal bias of small RNA production is conserved across angiosperms and is considered a key driver of imprinting evolution.

During embryogenesis, small RNAs maintain TE repression as the zygote transitions from gametic to somatic chromatin states. Embryos of *Arabidopsis*, rice, and maize accumulate high levels of 24-nt siRNAs, which guide CHH methylation at TE edges and prevent ectopic transcription during rapid cell division [86,103,104]. Loss of RdDM components leads to TE derepression, aberrant embryo patterning, and reduced seed viability, demonstrating that small RNA-mediated silencing is indispensable for early developmental robustness.

Seed development introduced additional selective pressures that shaped small RNA evolution. The endosperm, which undergoes extensive demethylation, is a major source of siRNAs that reinforce TE silencing in the embryo and contribute to maternal control over seed development [91,94]. Endosperm-derived siRNAs can move into the embryo, establishing a cross-tissue silencing system that stabilizes large, TE-rich genomes during seed maturation [105,106,107].

One unresolved evolutionary question concerns the origin of reproductive methylation reprogramming. In flowering plants, active DNA demethylation in companion cells generates epigenetic asymmetries between reproductive cell lineages and contributes to transposon silencing, imprinting, and seed development. However, it remains unclear whether these mechanisms represent angiosperm-specific innovations associated with double fertilization and endosperm evolution or whether they evolved from more ancient forms of reproductive chromatin resetting already present in early land plants. Comparative genomic analyses suggest that components of DNA methylation and demethylation machinery predate angiosperms, yet direct evidence for dynamic reproductive epigenetic reprogramming outside flowering plants remains limited.

Environmental stress has likely been an important selective force shaping the evolution of reproductive epigenetic regulation. Abiotic stresses, including heat, drought, and salinity, can alter DNA methylation, histone modifications, and small RNA pathways in reproductive tissues, thereby affecting tapetum function and pollen–pistil interactions, genomic imprinting during endosperm and embryo development, and the timing of seed maturation. Although these epigenetic responses are increasingly recognized as important components of reproductive plasticity, direct evidence that environmental stress modifies the epigenetic regulation of fertilization itself remains limited. Identifying the molecular targets of stress-induced epigenetic remodeling during gamete recognition, pollen tube reception, fertilization, and early seed development represents an important direction for future research. From an evolutionary perspective, stress-responsive epigenetic mechanisms may have provided a flexible regulatory layer that enhanced reproductive resilience under fluctuating environmental conditions while contributing to long-term adaption. This evolutionary plasticity may have contributed to the diversification of reproductive strategies across land plants.

### 4.6. Epigenetic Regulation of Seed Dormancy and Developmental Memory

Seed dormancy is one of the most consequential evolutionary innovations of seed plants, enabling embryos to survive seasonal unpredictability and disperse through time and space. The establishment, maintenance, and release of dormancy rely on a multilayered epigenetic framework that integrates hormonal signaling, chromatin state, and environmental cues [108,109]. Comparative studies across bryophytes, gymnosperms, and angiosperms suggest that the epigenetic mechanisms underlying dormancy and developmental memory in flowering plants were progressively assembled through the evolutionary co-option of ancestral stress-response and genome-defense pathways [23].

In angiosperms, dormancy is established during late embryogenesis through coordinated repression of germination-promoting genes and stabilization of abscisic acid (ABA) signaling. Central regulators such as *DOG1*, *ABI3*, and *LEC1/LEC2* are controlled by dynamic chromatin modifications, including H3K27me3 deposition by PRC2 and H3K9me2-associated heterochromatin [110]. PRC2-mediated repression of embryonic transcription factors is conserved across seed plants but its recruitment to dormancy loci appears to be an angiosperm-specific elaboration, reflecting the increasing complexity of seed maturation programs. In *Arabidopsis*, the transition from embryogenesis to dormancy is marked by a genome-wide shift toward repressive chromatin, with PRC2 and H3K27me3 acting as long-term stabilizers of the dormant state [6].

Although vernalization-associated regulation of *FLOWERING LOCUS C* (*FLC*) is typically discussed in the context of flowering time evolution, it also illustrates a broader seed-plant innovations in epigenetic developmental memory mediated by Polycomb-dependent H3K27me3 [111]. Such systems represent derived elaborations of older chromatin memory mechanisms that originally evolved in developmental phase regulation but were co-opted into seed ecological adaptation.

DNA methylation also contributes to dormancy by reinforcing transcriptional quiescence [112]. CHH methylation at TE-rich regions increases during seed maturation, driven by RdDM and CMT2-dependent pathways, providing genome stabilization during desiccation and long-term storage [112,113]. Comparative methylome analyses demonstrate that gymnosperm embryos maintain a consistently high global methylation level, particularly in CG and CHG contexts, throughout seed development, lacking the extensive, dynamic reprogramming (such as the drastic CHH methylation increase/decrease cycles) characteristic of angiosperms [81]. The evolution of active DNA demethylation mediated by DNA glycosylases such as *ROS1*, *DME*, *DML2* and *DML3* further enabled fine-scale modulation of dormancy-related genes, allowing seeds to integrate environmental signals such as temperature and photoperiod into epigenetic decision-making [112].

A defining feature of seed evolution is the capacity to store developmental memory, allowing seeds to “record” environmental conditions experienced during maturation and use this information to modulate germination timing. Vernalization-like memory systems in seeds rely on stable chromatin marks, particularly H3K27me3, which persist through desiccation and imbibition. For example, the temperature experienced during seed development influences the chromatin state of *DOG1*, thereby altering dormancy depth in the next generation [114]. These environmentally induced chromatin states are mitotically stable but reversible, enabling seeds to balance long-term stability with ecological responsiveness.

From an evolutionary perspective, the integration of PRC2-mediated repression, DNA methylation dynamics, and small RNA pathways transformed ancestral stress-response mechanisms into a coordinated regulatory system capable of maintaining embryonic quiescence over extended periods. Gymnosperms exhibit elements of this system—high methylation, stable heterochromatin, and ABA-dependent repression—but lack the dynamic chromatin remodeling and maternal small-RNA reinforcement that characterize angiosperm seeds. Thus, the sophisticated epigenetic architecture underlying dormancy and developmental memory in flowering plants reflects a cumulative evolutionary trajectory in which ancient genome-defense pathways were progressively repurposed to support the ecological and developmental resilience that characterize seed-plant success.

## 5. Evolution of Epigenetic Control of Apomixis

**Figure 2:**
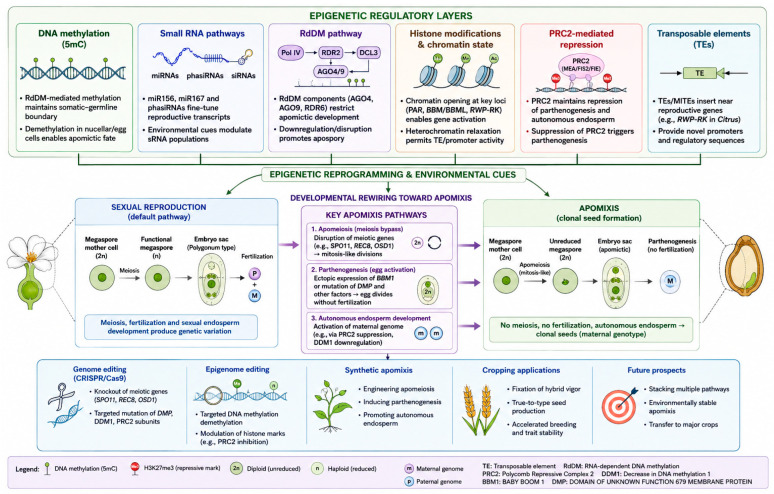
Conceptual diagram of epigenetic regulation of apomixis. The proposed model illustrates how epigenetic reprogramming redirects the canonical sexual reproductive pathway toward apomictic seed formation. In sexual reproduction (left), meiosis generates a reduced female gametophyte (n) that, following double fertilization, gives rise to the embryo (2n) and endosperm (3n). In gametophytic apomixis (right), three developmental transitions replace the sexual program: (i) apomeiosis, in which meiosis is bypassed to produce an unreduced embryo sac; (ii) parthenogenesis, whereby the embryo develops from an unfertilized diploid egg cell; and (iii) autonomous or pseudogamous functional endosperm development, depending on the species. The upper panel summarizes the principal epigenetic pathways implicated in this developmental switch. DNA methylation, RNA-directed DNA methylation (RdDM), small RNA pathways, chromatin remodeling, PRC2-mediated H3K27me3 repression, and transposable element (TE) regulation collectively maintain reproductive cell identity and repress embryogenesis and endospermogenesis before fertilization during sexual development. Alterations in these pathways, together with environmental and hormonal signals, promote developmental reprogramming by modifying chromatin accessibility and gene expression. Candidate regulators include components of the RdDM pathway (AGO, RDR, DCL), PRC2 complexes, DNA methylation machinery, and embryogenic regulators such as BABY BOOM (BBM/BBML). The lower panel highlights recent biotechnological strategies aimed at engineering synthetic apomixis. Genome editing, epigenome editing, and targeted manipulation of meiotic, embryogenic, and gene imprinting pathways have demonstrated that apomixis can be reconstructed through coordinated modification of conserved reproductive regulatory networks. These advances provide promising opportunities for clonal seed production, fixation of hybrid vigor (heterosis), and crop improvement. The diagram represents a conceptual synthesis of current knowledge rather than a species-specific regulatory pathway.

Apomixis, the production of clonal seeds without meiosis and fertilization, represents a derived modification of the sexual reproductive program that enables genetically identical progeny to be transmitted through seeds. In gametophytic apomixis, the sexual pathway is reprogrammed at three key stages: (i) avoidance of meiosis (apomeiosis), (ii) parthenogenetic embryo initiation, and (iii) autonomous or pseudogamous endosperm formation (Figure 2) [1,115,116]. Comparative phylogenetic and genetic evidence indicates that apomixis has evolved repeatedly and independently across angiosperms, primarily through the deregulation or epigenetic reprogramming of conserved sexual developmental pathways rather than through entirely novel genetic modules [117]. This recurrent origin identifies epigenetic regulation as a major source of reproductive plasticity, capable of redirecting canonical sexual development toward clonal reproduction. Beyond its evolutionary significance, apomixis has attracted considerable attention because of its enormous agricultural potential. The ability to fix superior hybrid genotypes through clonal seed production could revolutionize crop breeding by permanently preserving hybrid vigor (heterosis) and other desirable agronomic traits. Consequently, elucidating the molecular and epigenetic mechanisms underlying apomixis is essential not only for understanding the evolution and plasticity of plant reproductive systems but also for developing innovative strategies for sustainable crop improvement.

### 5.1. Epigenetic Reprogramming of the Sexual Pathway as a Basis for Apomixis

Current models converge on the idea that apomixis arises through the partial uncoupling or temporal misregulation of core reproductive processes, including meiosis (apomeiosis), fertilization (parthenogenesis), and endosperm formation, each of which is normally under tight epigenetic control. These processes are not replaced but reconfigured, supporting the view that apomixis represents a developmental reprogramming of sexuality mediated by altered chromatin states and transcriptional networks [118]. Epigenetic pathways are essential for germline specification and gametophyte development. Disruption of these pathways can induce apomixis-like phenotypes in otherwise sexual species, demonstrating that the sexual–apomictic switch is, at least in part, epigenetically encoded [119]. Recent advances further support the central role of epigenetic regulation in apomixis. Alterations in DNA methylation patterns, small RNA pathways, and Polycomb-mediated repression have been shown to influence the switch between sexual and apomictic development in several systems (Figure 2). Together, these observations indicate that apomixis is an emergent property of the coordinated deregulation of multiple epigenetic pathways rather than the consequence of a single master regulator.

Comparative analyses between sexual and apomictic taxa reveal consistent alterations in DNA methylation landscapes. In several apomictic systems (e.g., *Boechera*, *Hieracium*, *Paspalum*), shifts in global or locus-specific methylation are consistently associated with reproductive mode. Both hypo- and hypermethylation states have been associated with apomixis depending on lineage and developmental context, suggesting that precise spatial and temporal methylation control, rather than absolute levels, is critical [116,120].

Small RNA pathways appear particularly important in this transition. Differential expression of siRNA biogenesis and RdDM-related genes between sexual and apomictic plants indicates that epigenetic silencing of key reproductive regulators is modulated during the evolution of apomixis [121,122,123]. These pathways influence cell-fate specification within the ovule, determining whether somatic nucellar cells or meiotic products give rise to the embryo sac (apospory versus diplospory).

Endosperm development represents a major evolutionary constraint on apomixis. In sexual angiosperms, genomic imprinting and parent-of-origin-specific methylation patterns ensure balanced maternal and paternal contributions. In most angiosperms, normal endosperm development depends on a 2:1 maternal-to-paternal ratio (dosage effect) maintained through genomic imprinting and parent-of-origin-specific epigenetic regulation. Consistent with the parental conflict hypothesis discussed above, genomic imprinting contributes to balanced endosperm development by regulating parent-of-origin-specific gene expression. Apomictic species circumvent this requirement through lineage-specific modifications of imprinting networks [17,124]. In pseudogamous apomicts, paternal genome contribution is still required for endosperm, but imprinting is relaxed, allowing fertilization with genetically unrelated pollen. In autonomous apomicts, imprinting regulators such as MEA-PRC2 show reduced expression or altered chromatin states, enabling endosperm formation without paternal input. These lineage-specific modifications illustrate how genomic imprinting, a relatively recent angiosperm innovation, became a key evolutionary constraint on apomixis [17,125]. Partial relaxation or rewiring of imprinting networks therefore appears to have been a prerequisite for the repeated evolution of apomictic reproduction.

### 5.2. Apomixis-like Reproduction in Plant Lineages Outside Angiosperms

Apomixis *sensu stricto* refers to the production of seeds without meiosis and fertilization and is therefore an angiosperm-specific reproductive strategy. The term is often used interchangeably with agamospermy, both describing the production of genetically identical progeny through seeds. More broadly, however, analogous forms of asexual reproduction that bypass one or both stages of sexual reproduction occur in several other plant lineages. Although these processes operate through different developmental mechanisms and do not involve seed formation, they similarly produce offspring that are genetically identical or nearly identical to the maternal parent. From an evolutionary perspective, comparison of these reproductive strategies is informative because many of the same epigenetic regulatory modules—including DNA methylation, histone modifications, and small RNA pathways—control the key developmental transitions between meiosis, gametogenesis, and embryogenesis across the photosynthetic eukaryotes.

Apomixis-like reproductive strategies have been described in bryophytes, ferns, and some algal lineages, whereas they appear to be extremely rare in gymnosperms [126,127,128]. Although their underlying developmental mechanisms differ substantially from those of flowering plants, these systems provide valuable comparative models for understanding how conserved epigenetic pathways have been repeatedly co-opted during the evolution of asexual reproduction.

Apomixis-like reproduction is well-documented in brown algae (e.g., *Ectocarpales*, *Laminariales*), often taking the form of parthenogenesis, where an embryo develops from an unfertilized egg or apogamy, where a sporophyte develops directly from the vegetative cells of a gametophyte. However, recent studies suggest that brown algae lack several canonical epigenetic pathways that regulate reproductive development in angiosperms. In particular, DNA methylation and Polycomb Repressive Complex 2 (PRC2)-mediated repression appear to have been lost early during brown algal evolution [129,130]. Instead, brown algae have evolved alternative chromatin-based regulatory systems, including H3K79 methylation, to control gene expression [130]. These findings raise the possibility that parthenogenesis and other forms of asexual reproduction in brown algae are regulated by epigenetic mechanisms distinct from those operating in land plants. However, the molecular basis of these processes remains largely unknown.

In bryophytes (mosses, liverworts, hornworts) apomixis is rare but does occur, primarily in the form of apogamy, in which a diploid sporophyte develops directly from haploid or polyploid gametophytic cells without fertilization. This phenomenon has been observed particularly in older cultures, where diploid sporophyte-like structures can arise spontaneously from gametophyte cells [82,131]. Studies in the moss *Physcomitrium patens* have demonstrated that Polycomb Repressive Complex 2 components are essential preventing premature activation of the sporophytic developmental program in the gametophyte. Loss of PRC2 function, for example in *Ppclf* or *Ppfie* mutants, leads to ectopic activation of sporophyte-specific genes and the formation of apogamous sporophyte-like bodies on the gametophyte without fertilization [33,41]. This developmental switch is mediated by PRC2 components, including the orthologs *PpCLF* (*CURLY LEAF*) and *PpFIE* (*FERTILIZATION-INDEPENDENT ENDOSPERM*), which maintain H3K27me3-dependent repression of sporophytic regulators during the gametophyte stage. Loss of this repression leads to derepression of key developmental regulators, including the *BELL*-type homeodomain transcription factors *PpBELL1* and *PpBELL2***,** thereby initiating sporophyte development independently of fertilization. Although the molecular mechanisms differ from those operating in flowering plants, these findings illustrate a common evolutionary principle: disruption of epigenetic repression can uncouple sporophyte development from fertilization, suggesting that Polycomb-mediated chromatin regulation represents a conserved mechanism controlling reproductive phase transitions across land plants [33,41].

Ferns (Pteridophytes) provide the clearest non-seed-plant analogs of apomixis. Many species exhibit apogamy (sporophyte formation from gametophyte cells without fertilization) and apospory (gametophyte formation from sporophytic tissues without meiosis). These processes effectively bypass canonical alternation of generations and are often associated with polyploidy, particularly in the families Pteridaceae (e.g., *Pteris*) and Dryopteridaceae, paralleling patterns observed in angiosperm apomixis. Apomixis has been reported in approximately 10% of fern species [132,133]. Apogamy can be induced by environmental cues, including changes in light conditions, carbohydrate availability, and water stress, and accumulating evidence suggests that these responses are mediated, at least in part, through epigenetic regulation [122,134]. Environmental signals can alter gene expression by modifying DNA methylation and chromatin organization, thereby promoting the transition from sexual to apogamous development. Although the underlying molecular mechanisms remain less well characterized than in angiosperms, DNA methylation and chromatin modifications appear to contribute both to developmental reprogramming and to the maintenance of genome stability through transposable element (TE) silencing. Moreover, apogamy can also be induced experimentally by exogenous application of plant growth regulators such as naphtaleneacetic acid (NAA) and gibberellic acid (GA), which are thought to influence epigenetic regulation of developmental pathways [122,135,136]. Collectively, these findings suggest that ferns represent an important evolutionary intermediate in which environmentally responsive epigenetic mechanisms regulate the switch between sexual and asexual reproduction, providing insights into the evolutionary origins of apomixis in land plants.

In gymnosperms, reproduction is strictly seed-based and predominantly sexual, involving well-defined male and female gametophytes. Reports of apomixis are extremely rare and often ambiguous, with most cases attributable to polyembryony (the development of multiple embryos within a single seed), a common feature of gymnosperms, rather than true fertilization-independent development (apomixis) [137,138]. A rare case of apomixis (androgenesis) has been observed in *Cupressus*, although its molecular basis remains poorly understood [139]. Nevertheless, several aspects of gymnosperm reproduction provide important insights into the evolutionary context of apomixis. Gymnosperm genomes are exceptionally large and rich in transposable elements (TEs), creating strong selective pressure for robust epigenetic silencing mechanisms. Accordingly, DNA methylation and small RNA pathways are highly active, particularly during reproductive development. Dynamic changes in DNA methylation and histone modifications accompany megagametophyte development and embryogenesis, reflecting extensive chromatin remodeling during these developmental transitions [11,81,140]. However, unlike angiosperms, gymnosperms show little evidence for genomic imprinting or for epigenetically regulated fertilization-independent embryogenesis [141,142]. This contrast suggests that although the core epigenetic machinery controlling genome stability is evolutionarily conserved, its recruitment into specialized reproductive developmental pathways associated with apomixis occurred primarily during the evolution of flowering plants.

Experimental perturbations of hormone signaling and exposure to stress conditions can induce somatic embryogenesis in gymnosperms, a process widely exploited in clonal forestry [143,144]. These observations demonstrate that embryogenic competence can be epigenetically reactivated in somatic cells even though natural apomixis is absent. From an evolutionary perspective, this suggests that the developmental potential for clonal embryo formation is present but has not been naturally canalized, possibly because of constraints associated with gymnosperm reproductive biology, including the absence of endosperm and the tighter developmental coupling between fertilization and embryo development.

### 5.3. Recent Molecular Advances and Biotechnological Applications

Recent advances have substantially refined the molecular understanding of apomixis, demonstrating that this reproductive mode does not arise as an independent developmental program but rather through epigenetic reprogramming of conserved sexual pathways (Figure 2). Comparative genomic and epigenomic studies show that apomixis-associated loci are frequently enriched in transposable elements and located within heterochromatic regions, suggesting that local chromatin architecture plays a central role in determining reproductive fate. In several systems, including *Taraxacum*, *Citrus*, *Paspalum*, and *Boechera*, insertion of transposable elements or miniature inverted-repeat transposable elements (MITEs) near key developmental genes has been shown to alter their regulation, effectively rewiring sexual developmental pathways [1,117,145]. These observations support the view that the repeated, independent origins of apomixis across angiosperms are primarily driven by changes in epigenetic regulation rather than by the evolution of novel genes.

A central mechanistic insight is the key role of RNA-directed DNA methylation (RdDM) and small RNA pathways in establishing and maintaining reproductive identity. Disruption or downregulation of RdDM components, including *ARGONAUTE* (*AGO4*, *AGO9*) and *RNA-DEPENDENT RNA POLYMERASE* (*RDR6*), leads to a breakdown of the somatic–germline boundary and can induce the formation of unreduced female gametophytes through apospory or diplospory [120,123,146]. Importantly, this transition depends not on global epigenetic resetting but on selective, locus-specific modifications of DNA methylation and chromatin accessibility, which allow reprogramming of key regulatory networks. PRC2 plays a crucial role in maintaining the repression of fertilization-independent embryogenesis (parthenogenesis) and endosperm development; reduction of PRC2 activity or alteration of imprinting states can activate parthenogenesis and autonomous seed formation [120,123,146].

Additional regulatory layers are provided by small non-coding RNAs. MicroRNAs (e.g., miR156, miR167) and phased small interfering RNAs (phasiRNAs) fine-tune the expression of reproductive genes and link epigenetic regulation to environmental signals. Variation in the profiles of these RNAs can influence the transition between sexual and apomictic reproduction, particularly under stress conditions [121,147]. Moreover, changes in chromatin accessibility at key regulators of embryogenesis, such as *PAR* or *BBML*, enable activation of developmental programs in the absence of fertilization (Figure 2) [148,149].

From a translational perspective, these discoveries have driven significant progress toward the engineering of “synthetic apomixis” by mimicking natural apomixis via epigenome editing and CRISPR/Cas9. Modern approaches combining genome editing with epigenetic manipulation can reproduce essential components of apomictic development in model and crop species. Replacement of meiosis with mitosis-like divisions has been achieved through modification of meiotic genes such as *SPO11-1*, *REC8* or *OSD1*, thereby preventing chromosome reduction [1,147,150]. Concurrently, induction of fertilization-independent embryogenesis can be triggered by ectopic expression of embryogenic regulators such as *BABY BOOM1 (BBM1)* or by mutation of haploid-inducer genes such as *DMP* (Figure 2) [117,150].

Epigenetic regulation remains a critical component of these systems. Manipulation of DNA methylation, chromatin structure, and small RNA pathways strongly influences the efficiency and stability of engineered apomixis, indicating that successful implementation will require coordinated modification of both genetic and epigenetic regulators. Although stable and agriculturally deployable apomixis has not yet been achieved, current advances demonstrate that the underlying regulatory architecture is experimentally accessible [123,151].

Collectively, these findings support the hypothesis that apomixis evolved primarily through repeated epigenetic rewiring of an ancestral sexual reproductive program rather than through the origin of fundamentally new developmental pathways. This evolutionary plasticity explains both the multiple independent origins of apomixis in angiosperms and its considerable potential for biotechnological manipulation.

## 6. Future Directions and Research Gaps

Despite substantial progress in understanding the evolution of epigenetic regulation in plant reproduction, several fundamental questions remain unresolved. Addressing these gaps will be essential for developing a coherent evolutionary framework and for translating epigenetic knowledge into practical applications.

A primary unresolved issue concerns the evolutionary origin of reproductive epigenetic reprogramming (Table 2). While extensive, cell type-specific DNA methylation remodeling is well documented in angiosperms, particularly in relation to double fertilization and endosperm development, similar dynamics are largely absent in gymnosperms and bryophytes. Why dynamic epigenetic reprogramming evolved in flowering plants despite comparable, and in some cases greater, genome complexity in other lineages remains unclear. Comparative studies integrating developmental biology, genome architecture, and functional epigenomics across non-model taxa will be essential to determine when and why these specialized regulatory programs emerged. Such studies should also clarify how plants maintain epigenetic homeostasis while permitting localized chromatin remodeling during key reproductive transitions. Reproductive development requires maintenance of genome integrity across generations while simultaneously allowing localized epigenetic reprogramming in specific cell types. Understanding how this balance is achieved remains a major challenge.

A second major gap relates to the conservation versus divergence of epigenetic mechanisms across plant lineages. Core components such as DNA methylation machinery, small RNA pathways, and Polycomb complexes are deeply conserved, yet their reproductive functions vary substantially (Table 2). It remains unclear to what extent these functions are evolutionarily constrained or represent repeated, lineage-specific co-option events. In particular, genomic imprinting, although central to angiosperm development, shows strong variability across species, suggesting that it may arise convergently from shared epigenetic modules rather than from a single evolutionary origin.

A third critical area involves the role of epigenetic regulation in reproductive adaptation under environmental change. While epigenetic mechanisms are known to mediate stress responses and developmental plasticity, their contribution to reproductive fitness, transgenerational inheritance, and long-term adaptation remains insufficiently resolved. In natural populations, distinguishing adaptive epigenetic variation from transient or neutral changes is a major challenge. Understanding how epigenetic states influence reproductive success under fluctuating environments will be particularly important in the context of rapid climate change.

Addressing these questions will increasingly depend on emerging technologies. Single-cell and spatial epigenomics, long-read methylome sequencing, chromatin accessibility profiling, and comparative epigenomic analyses are beginning to resolve epigenetic dynamics at cellular resolution across diverse plant lineages. Together with CRISPR/dCas-based epigenome editing and experimental evolution approaches, these methods will enable direct testing of the causal roles of epigenetic modifications in reproductive development and their evolutionary significance.

From a translational perspective, these advances hold significant potential for crop improvement and sustainable agriculture. Targeted manipulation of epigenetic pathways could enable the control of flowering time, fertility, hybrid vigor, seed dormancy, and apomixis-based clonal seed production. Integrating evolutionary insights with applied epigenetics may therefore provide novel strategies to enhance crop resilience and productivity under changing environmental conditions.

Overall, future research should aim to bridge the gap between evolutionary theory, developmental biology, and applied plant sciences. Such integration will be critical for fully understanding how epigenetic systems have shaped plant reproductive diversity and how they can be harnessed to meet global agricultural challenges.

## 7. Conclusions

The evolution of epigenetic regulation in plant reproduction is best understood as a process of functional co-option and regulatory rewiring of an ancient molecular toolkit rather than the gradual accumulation of entirely new regulatory pathways. Epigenetic mechanisms originated early in eukaryotic evolution and were subsequently retained throughout the green lineage. During plant evolution, these conserved mechanisms were progressively integrated into reproductive development, acquiring increasingly specialized roles in gametophyte differentiation, fertilization, embryogenesis, and seed formation.

A central conclusion emerging from this synthesis is that deep molecular conservation coexists with extensive functional diversification. Although the core epigenetic machinery has remained remarkably stable, its deployment has been repeatedly reorganized during plant evolution. This trend reached its greatest complexity in angiosperms, where epigenetic regulation became closely associated with double fertilization, endosperm development, genomic imprinting, and communication between reproductive cell types. In contrast, bryophytes and gymnosperms retain comparatively stable epigenetic landscapes, indicating that extensive reproductive epigenetic reprogramming represents a derived feature of flowering plants rather than a universal characteristic of land plant reproduction.

The repeated evolutionary recruitment of conserved epigenetic pathways also demonstrates that plant reproductive systems remain intrinsically flexible. The independent emergence of apomixis and other alternative reproductive strategies illustrates how relatively modest modifications of DNA methylation, chromatin regulation, and small RNA pathways can redirect developmental programs without fundamentally altering the underlying genetic framework. Epigenetic regulation therefore functions both as a mechanism ensuring genome stability and as a source of developmental and evolutionary innovation.

Overall, plant reproductive epigenetics should be viewed not as a secondary layer superimposed on genetic regulation, but as a dynamic and evolvable regulatory system integrating genome defense, development, and environmental responsiveness. Through repeated co-option, diversification, and functional specialization of conserved molecular pathways, epigenetic mechanisms have played a central role in the origin of reproductive innovations and the evolutionary success of land plants.

## Figures and Tables

**Table 1:** Functional roles of core epigenetic gene families across major plant evolutionary lineages.

	Gene Family	*AGO*	*DCL*	*RDR*	*CMT*	*DRM*	*PRC2*	*DDM1*
Plant Lineage								
Streptophyte algae		Present (ancestral AGO clades; basic RNA silencing)	Present (simplified miRNA/siRNA processing)	Present (early RNA amplification)	Rare/incipient homologs (limited CHG methylation)	Present (de novo methylation machinery emerging)	Present (primitive PcG system; developmental repression)	Present (basic heterochromatin remodeling)
Bryophytes		Expanded AGO families; gametophyte regulation, TE silencing	Multiple DCLs; miRNA/siRNA diversification	Functional RDRs; tasiRNA pathways active	Conserved CMT homologs; CHG maintenance methylation	DRM-mediated RdDM established	Functional PRC2; repression of sporophyte programs in gametophyte	Functional; TE silencing and chromatin compaction
Lycophytes		Diversified AGO clades; reproductive and meristem regulation	DCL diversification similar to seed plants	RDR-dependent siRNA amplification conserved	Conserved CMTs; heterochromatin methylation	RdDM functional; TE silencing	PRC2 conserved; developmental phase regulation	Conserved; heterochromatin accessibility control
Ferns		AGO expansion; roles in apogamy/phase transitions (inferred)	DCL pathways conserved; reproductive regulation	RDRs active; siRNA pathways in development	CMT homologs shared with seed plants; CHG/CHH methylation	DRM-dependent RdDM conserved	PRC2 conserved; phase identity control	Conserved; chromatin remodeling in large genomes
Gymnosperms		Expanded AGO families; TE silencing in large genomes	DCL diversification; reproductive small RNAs	RDR pathways active; siRNA amplification	CMT homologs (CMT2/3-like); gene-body methylation-like patterns	DRM functional; RdDM in reproductive tissues	PRC2 conserved; embryogenesis regulation	Highly active; TE silencing in large, repeat-rich genomes
Basal angiosperms		AGO diversification; germline and imprinting roles emerging	Full DCL complement; reproductive siRNAs	RDR expansion; tasiRNA and siRNA pathways	CMT diversification; emergence of gene-body methylation (gbM)	DRM robust; canonical RdDM	PRC2 central to seed development and imprinting	Essential for heterochromatin maintenance
Monocots		Expanded AGO clades; reproductive phasiRNAs	DCL specialization (e.g., DCL5 for reproductive siRNAs)	RDR diversification; phasiRNA pathways	CMT variants (e.g., ZMETs); lineage-specific functions	DRM active; RdDM with lineage-specific features	PRC2 conserved; endosperm regulation	Strong TE silencing in large genomes
Eudicots		Highly diversified AGO family; germline specification, RdDM	DCL1–4 specialization; miRNA/siRNA partitioning	RDR1/2/6 specialization; tasiRNA, antiviral roles	CMT1/2/3 diversification; gbM dependent on CMT3	DRM2 central to RdDM; epigenetic regulation of reproduction	PRC2 highly specialized; imprinting, embryo/endosperm patterning	Essential chromatin remodeler for TE silencing

**Table 2:** Comparative overview of epigenetic regulation in plant reproduction across major lineages.

Mechanism	Streptophyte Algae	Bryophytes	Ferns	Gymnosperms	Angiosperms
DNA methylation	✓	✓	✓	✓	✓
Canonical RdDM	–	partial	partial	✓	✓
Active DNA demethylation	✓	✓	✓	✓	✓
Imprinting	–	limited	–	–	✓
Reproductive phasiRNAs	–	–	–	✓	✓
Reprogramming	✓	✓	✓	✓	Extensive
Small RNA specialization	✓	✓	✓	✓	Extensive

The distribution and relative complexity of major epigenetic mechanisms associated with plant reproduction vary across representative green plant lineages. Check marks (✓) indicate the presence of a mechanism, “partial” denotes incomplete or lineage-specific pathways, “limited” indicates restricted evidence or occurrence, and “–” indicates no convincing evidence currently available. “Extensive” denotes pronounced functional specialization and dynamic regulation, particularly in angiosperm reproduction.

## Data Availability

No new data were created or analyzed in this study.
